# Plasma biomarkers are associated with agitation and regional brain atrophy in Alzheimer’s disease

**DOI:** 10.1038/s41598-017-05390-1

**Published:** 2017-07-11

**Authors:** Jung-Lung Hsu, Wei-Ju Lee, Yi-Chu Liao, Jiing-Feng Lirng, Shuu-Jiun Wang, Jong-Ling Fuh

**Affiliations:** 1 Taipei Medical University, Graduate Institute of Humanities in Medicine, Taipei, Taiwan; 2 Department of Neurology, Chang Gung Memorial Hospital Linkou Medical Center and College of Medicine, Chang-Gung University, Taoyuan, Taiwan; 3 Taipei Medical University Research Center for Brain and Consciousness and Shuang Ho Hospital, New Taipei City, Taiwan; 4 Neurological Institute, Taichung Veterans General Hospital, Taichung, Taiwan; 5 Faculty of Medicine, National Yang-Ming University Schools of Medicine, Taipei, Taiwan; 6 Institute of Clinical Medicine, National Yang-Ming University Schools of Medicine, Taipei, Taiwan; 7 Brain Research Center, National Yang-Ming University Schools of Medicine, Taipei, Taiwan; 8 Department of Neurology, Neurological Institute, Taipei veterans General Hospital, Taipei, Taiwan; 9 Department of Radiology, Taipei Veterans General Hospital, Taipei, Taiwan

## Abstract

This study investigated the relationships among plasma biomarkers, regional brain atrophy, and clinical symptoms in patients with Alzheimer’s disease (AD; n = 177), mild cognitive impairment (MCI; N = 60) and controls (n = 108). The Mini-Mental Status Examination (MMSE), Clinical Dementia Rating (CDR), and Neuropsychiatric Inventory (NPI) subscales were administered to subjects. Magnetic resonance imaging was performed and medial temporal atrophy (MTA) and posterior atrophy (PA) were assessed visually. We examined associations among cognition, NPI score, plasma β-amyloid (Aβ) and clusterin levels, and regional brain atrophy in patients with AD by regression analysis. The mean MTA score was associated with the plasma Aβ1-42/Aβ1-40 ratio (r = 0.38, p = 0.01) and with MMSE scores (r = 0.43, p < 0.01). The plasma clusterin level was correlated with CDR sum of box and right-side PA scores (r = 0.28, p = 0.01 and r = 0.30, p = 0.03, respectively). Right-side PA scores were correlated significantly with NPI agitation/aggression (r = 0.30, p = 0.03) subscale scores. In conclusion, the plasma ratio of Aβ1-42/Aβ1-40 and clusterin level may be associated with different patterns of regional brain atrophy, which in turn may account for the clinical symptoms in patients with AD.

## Introduction

Alzheimer’s disease (AD) is a neurodegenerative disorder characterized by progressive cognitive dysfunction, behavioral changes, and loss of functional ability. The diagnosis of AD relies on the presence of cognitive impairment, namely, episodic memory loss combined with other cognitive deficits. Several cognitive screening tests, including the Mini-Mental State Examination (MMSE) and the DemTect^1, 2^, have been developed to measure the cognitive deficits in AD. These deficits are correlated with structural changes in the brain^3, 4^. In addition, 75–90% of patients with AD develop “non-cognitive” behavioral and psychological symptoms of dementia (BPSD), which can be classified using the Neuropsychiatric Inventory (NPI)^5–8^. The prevalence of BPSD in patients with early-stage AD ranges from 17% to 46%. Factor analysis can be used to cluster these symptoms into several behavioral groups^6, 9^, typically consisting of three or four of the following: agitation/aggression (hyperactivity), mood/apathy, frontal symptoms, and psychosis^8, 9^. Agitation and aggression symptoms are difficult to manage and are especially distressing for patients, caregivers, and medical staff members. The underlying pathophysiological mechanisms of BPSD are not understood, although genetic factors (e.g., apolipoprotein E [ApoE] polymorphism) and certain regional brain dysfunctions have been suggested to play significant roles^10–12^.

Newly developed non-invasive neuroimaging tools, such as brain magnetic resonance imaging (MRI), aid the examination of relationships between cognitive and behavioral symptoms and regional brain dysfunction. From an anatomical point of view, regional brain atrophy is associated with different cognitive symptoms^13^. In BPSD, gray matter loss in the frontolimbic cortex has been associated with agitation/aggression symptoms^12^. Atrophy of the lateral frontal, parietal, and anterior cingulate gyrus has been associated with the onset of psychotic symptoms, including delusion, agitation, wandering, and hallucination, and/or the need for chronic use of antipsychotic medication^14^. In addition, cerebral white matter lesions have been associated independently with BPSD in patients with AD^15^. Although various brain regions are associated with agitation and aggression symptoms, their relationships to regional brain changes and plasma biomarkers remain unknown.

Plasma amyloid ß peptide (Aß) is a blood-based biomarker used to detect underlying AD pathology^16^. Increased and decreased plasma amyloid ß peptide 1–40 (Aß1-40) and amyloid ß peptide 1–42 (Aß1-42) levels have been reported in patients with AD^17–19^. Increased plasma Aß1-40 levels have been associated with atrophy of the medial temporal lobe structures in subjects without dementia^20^. Plasma amyloid levels have also been correlated with cognitive dysfunction in patients with AD^21^. Plasma clusterin, also known as apolipoprotein J, is a chaperone protein that is expressed widely in brain tissue. This plasma biomarker may also be related to the diagnosis of AD and regional brain atrophy^22^. In our previous study, right-side posterior atrophy (PA) and medial temporal atrophy (MTA), measured by MRI, were associated with the NPI agitation/aggression subscale score and cognitive impairment in patients with AD^11^. The current study was conducted to further investigate the relationships among plasma Aß and clusterin levels, atrophy ratings determined by MRI assessment, and clinical symptoms in patients with AD and those with mild cognitive impairment (MCI).

## Results

### Demographic, clinical and plasma biomarker data from study subjects

A total of 345 patients (probable AD, n = 177; MCI, n = 60, control, n = 108) were recruited during the study period. The mean (±standard deviation) ages of patients with AD were 81.0 ± 5.8 and 73.9 ± 8.3 years for patients with MCI and 74.6 ± 7.7 years for controls; 49.2% of patients with AD, 45.2% of patients with MCI and 44.4% controls were women (Table 1). In the AD group, mean NPI agitation/aggression, mood, and frontal symptoms scores were 6.8, 5.5, and 5.5, respectively. Values for plasma Aß1-40 and Aß1-42 levels and the Aß1-42/Aß1-40 ratio were log transformed for further analysis due to the high degree of kurtosis (>3) of data distribution. Plasma Aß1-40 and clusterin levels showed a significant group difference between patients with AD and controls and patients with MCI and controls by t-tests (all p < 0.01; Table 2). In a regression analysis adjusted for age, sex, and ApoE4 carrier status, the significant higher level of in plasma Aß1-40 and clusterin levels were found in patients with AD and MCI than controls (all p < 0.01, respectively). The body mass index (BMI) was not associated with the plasma Aß1-40, Aß1-42, or clusterin level or the Aß1-42/Aß1-40 ratio in the adjusted analysis.

**Table 1:** Demographic and clinical data for the whole sample and patients with AD, MCI and controls.

	AD (n = 177)	MCI (n = 60)	Controls (n = 108)	*p* value^#^	*p* value^&^
Female patients	87 (49.2%)	28 (45.2%)	48 (44.4%)	0.44	0.78
Age	81.0 (5.8)	73.9 (8.3)	74.6 (7.7)	<**0**.**0001**	**0**.**60**
Education (years)	10.1 (4.3)	10.8 (4.7)	10.3 (5.1)	0.62	0.50
Disease duration (years)	3.0 (3.6)	1.8 (1.7)	—		
BMI	23.7 (3.4)	23.9 (3.4)	24.3 (3.1)	0.16	0.50
MMSE	19.4 (4.9)	25.6 (2.8)	27.5 (2.5)	<**0**.**0001**	<**0**.**0001**
Delayed recall of 12-item memory test	1.4 (1.9)	4.4 (2.7)	—		
Forward digit span	8.7 (2.8)	9.7 (2.4)	—		
Backward digit span	4.1 (1.9)	5.3 (1.9)	—		
Category verbal fluency	6.9 (2.9)	9.5 (2.7)	—		
Modified Boston naming test	11.9 (2.2)	13.8 (1.1)	—		
NPI agitation/aggression subscale	6.8 (10.7)	1.5 (4.2)	—		
NPI mood	5.5 (8.5)	1.9 (4.5)	—		
NPI frontal symptoms	5.5 (8.8)	1.3 (3.8)	—		
CDR					
0.5	25 (14.1%)	60 (100.0%)	—		
1	120 (67.8%)				
2	29 (16.4%)				
3	3 (1.7%)				
ApoE4 carrier	64 (36.2%)	15 (25.0%)	17 (16.0%)	**0**.**0001**	**0**.**15**

Values are means with standard deviations unless otherwise indicated. Values in boldface indicate statistically significant differences between groups.

AD, Alzheimer’s dementia; MCI, mild cognitive impairment; BMI, body mass index; MMSE, Mini-Mental State Examination; NPI, Neuropsychiatric Inventory; CDR, clinical dementia rating.

^#^Compare AD and controls by two-sample t-tests; ^$^compare MCI and controls by two-sample t-tests.

**Table 2:** Plasma biomarkers for the whole sample and patients with AD, MCI and controls.

	AD (n = 177)	MCI (n = 60)	Controls (n = 108)	*p* value^#^	*p* value^&^
Aß1-40 (pg/ml)	170.3 (63.9)	171.1 (54.5)	143.7 (34.9)	**0**.**0001**	**0**.**0013**
Aß1-42 (pg/ml)	37.2 (14.1)	34.9 (9.5)	33.6 (10.2)	0.025	0.38
Ratio of Aß1-42/Aß1-40 (%)	23.2 (9.5)	21.0 (6.6)	23.9 (6.4)	0.14	0.0032
Clusterin (ug/ml)	248.6 (38.9)	256.5 (35.3)	232.3 (33.8)	**0**.**0005**	<**0**.**0001**

Values are means with standard deviations unless otherwise indicated.

AD, Alzheimer’s dementia; MCI, mild cognitive impairment; Aß, amyloid ß peptide.

^#^Compare AD and controls by two-sample t-tests; ^$^compare MCI and controls by two-sample t-tests.

### Plasma biomarkers, MRI visual ratings, and clinical symptoms

In patients with AD, the plasma Aß1-40 and clusterin levels were correlated significantly with age (Pearson correlation coefficient = 0.21 and −0.17, respectively, all p < 0.05). The plasma Aß1-42/Aß1-40 ratio was not associated with age, sex, disease duration, BMI, or ApoE4 carrier status, but it was associated weakly with the mean MTA score (Pearson correlation coefficient = −0.16, p = 0.04; Table 3). The clusterin level was associated significantly with the Clinical Dementia Rating sum of box (CDR-SB) score (Pearson correlation coefficient = 0.14, p = 0.04). Plasma Aß1-40 and clusterin levels were not associated with the MMSE score or delayed recall of 12 items memory test.

**Table 3:** Correlations of plasma biomarker, demographics, disease characteristics and brain MRI visual rating scores in patients with AD.

	Aß1-40	Aß1-42	Ratio of Aß1-42/Aß1-40	Clusterin level
Age	0.21*	0.06	−0.13	−0.17*
Gender	−0.01	−0.13	−0.12	0.09
Disease Duration	−0.06	0.00	−0.02	0.05
ApoE4 carrier	0.00	−0.13	−0.13	0.06
MMSE	0.04	0.13	0.05	−0.04
CDR-SB	−0.08	−0.06	0.03	0.15*
MTA	0.12	−0.05	−0.16*	−0.09
PA	−0.06	0.15	0.14	0.07
FWMH	0.00	−0.08	−0.08	−0.08
POWMH	−0.05	−0.03	−0.02	−0.01
TWMH	−0.00	0.03	0.01	−0.05
TOWMH	−0.04	−0.03	−0.01	−0.05

Values are Pearson correlation coefficients.

MRI, magnetic resonance imaging; AD, Alzheimer’s disease; Aß, amyloid ß peptide; ApoE4, apolipoprotein E4; MMSE, Mini-Mental State Examination; CDR-SB, clinical dementia rating sum of boxes; MTA, medial temporal atrophy; PA, posterior atrophy; FWMH, frontal white matter hyperintensity; POWMH, parieto-occipital white matter hyperintensity; TWMH, temporal white matter hyperintensity; TOWMH, total white matter hyperintensity.

*p < 0.05.

A regression analysis adjusted for age, years of education, sex, and ApoE4 carrier status showed that the Aß1-42/Aß1-40 ratio was moderately significantly correlated with the mean MTA score (r = 0.38, p = 0.01), but not with the PA score, in patients with AD (Table 4). Post-hoc calculation of the statistical power achieved yielded a value of 0.99 under the conditions α = 0.05 and sample size = 170. The Aß1-42/Aß1-40 ratio was not associated with the MMSE score (statistical power = 0.90 under the same conditions). The clusterin level was correlated significantly with the CDR-SB and right-side PA scores after adjustment for multiple covariates (r = 0.28, p < 0.01 and r = 0.30, p = 0.03, respectively; Table 5). It was not correlated with the MMSE, agitation/aggression, frontal symptoms, or mean MTA score (statistical power >0.9 under the conditions α = 0.05 and sample size = 174). No significant association was observed between plasma biomarkers and the white matter hyperintensity (WMH) score. All post-hoc values for the statistical power achieved exceeded 0.80 under the conditions α = 0.05 and sample size = 176. In patients with MCI, no association was found between plasma biomarker, MMSE scores and NPI symptoms.

**Table 4:** Summary of regression analyses for ratio of Aß1-42/Aß1-40 predicting digit reversal and MTA in patients with AD.

Variables entered	R	R^2^	R^2^ Change	F Change	df
Digit reversal as dependent variable					
Block 1. Age, years of education, gender and ApoE4 carrier	0.16	0.03	0.03	1.47	165
Block 2. Ratio of Aß1-42/Aß1-40*	0.36	0.13	0.10	17.95*	164
MTA as dependent variable					
Block 1. Age, years of education, gender and ApoE4 carrier	0.33	0.11	0.11	5.06*	166
Block 2. Ratio of Aß1-42/Aß1-40*	0.38	0.15	0.04	7.05*	165

AD, Alzheimer’s dementia; CDR-SB, clinical dementia rating sum of boxes; ApoE, apolipoprotein E; Aß, amyloid ß peptide. *p < 0.05.

**Table 5:** Summary of regression analyses for clusterin level predicting the CDR-SB and PA (right side) in patients with AD.

Variables entered	R	R^2^	R^2^ Change	F Change	df
CDR-SB as dependent variable					
Block 1. Age, years of education, gender and ApoE4 carrier	0.21	0.04	0.04	1.86	170
Block 2. clusterin*	0.28	0.08	0.03	6.23*	169
PA (right side) as dependent variable					
Block 1. Age, years of education, gender and ApoE4 carrier	0.26	0.07	0.07	3.01	170
Block 2. Clusterin*	0.30	0.09	0.03	4.56	169

Aß, amyloid ß peptide; AD, Alzheimer’s dementia; MTA, medial temporal atrophy visual rating score; PA: posterior atrophy visual rating score; ApoE, apolipoprotein E.

*p < 0.05.

### MRI rating and clinical measurements in patients with AD

In patients with AD, MTA and PA scores within each hemisphere were not correlated (right side: r = 0.08, p = 0.32; left side: 0.10, p = 0.20). Regression analysis with adjustment for age, years of education, sex, and ApoE4 carrier status showed that the mean MTA score was moderately significantly correlated with the MMSE score in patients with AD (r = 0.43, p < 0.01). The post-hoc calculation of the statistical power achieved yielded a value of 0.99 under the conditions α = 0.05 and sample size = 177. We also observed a significant association between the CDR-SB and mean MTA scores (r = 0.30, p < 0.01). The mean PA score was not correlated significantly with the MMSE score after adjustment for age and years of education (statistical power = 0.89 under the conditions α = 0.05 and sample size = 176). The right-side PA score, but not the left-side PA or MTA score, was moderately significantly associated with the NPI agitation/aggression (r = 0.30, p = 0.03) and frontal symptoms (r = 0.31, p < 0.01) subscale scores after adjustment for multiple covariates. All post-hoc values for the statistical power achieved exceeded 0.90 under the conditions α = 0.05 and sample size = 176. In patients with MCI, no association was found between MRI visual rating scores, MMSE scores and NPI symptoms.

## Discussion

In this study, we investigated the relationships among plasma biomarkers, cognitive function, agitation/aggression symptoms, frontal symptoms, and MRI-based visual ratings in patients with AD and those with MCI. First, our results demonstrated that the plasma clusterin level was correlated with the severity of AD, as measured by the CDR-SB scale, even after adjustment for multiple covariates. Second, the plasma Aß1-42/Aß1-40 ratio was correlated with the mean MTA score, and the clusterin level was correlated with the right-side PA score, in patients with AD. Third, regional brain atrophy, as measured by the MTA score, was associated with MMSE and CDR-SB scores, and the right-side PA score was associated with the NPI agitation/aggression and frontal symptoms scores. These results suggest that different plasma biomarkers were linked individually to regional brain atrophy and that these changes were correspondingly associated with clinical symptoms in patients with AD.

### Plasma Aß levels, cognition, and brain structure

In patients with AD, increased plasma Aß1-40 or Aß1-42 levels have been reported^23, 24^. Other studies have shown no difference or even decreases in the Aß1-42 level^21, 25^. This wide variation in plasma Aß levels and the use of different assay techniques among studies have contributed substantially to discrepancies among findings^26^. In general, the broad overlap in plasma Aß levels between patients with AD and controls indicates that these levels cannot differentiate cases of sporadic AD from control cases. High plasma concentrations of Aß1-40 and low plasma concentrations of Aß1-42 indicate an increased risk of dementia^27^. In some studies, plasma Aß levels and the Aß1-42/Aß1-40 ratio have been associated significantly with MMSE and memory scores^21, 28^. However, other studies have found no association between plasma Aß levels and MMSE scores, leading the authors to conclude that age, rather than the AD diagnosis, was the main predictor of these levels^29^. These findings are consistent with our results: although the plasma Aß1-40 level of patients with AD was significantly higher than that of controls, even after adjustment for multiple covariates, the plasma Aß1-40 level and the Aß1-42/Aß1-40 ratio were not associated with the MMSE score. Our statistical power calculation confirmed that our sample size was sufficient to support the validity of these findings.

Findings regarding the relationships of plasma Aß levels to brain structure parameters are more consistent. In dementia-free subjects, a high plasma Aß1-40 level and low plasma ratio of Aß1-42/Aß1-40 were associated with smaller hippocampal volume and accelerated global and regional brain atrophy, respectively^20^. Sotolongo-Grau *et al*.^30^ also reported that the left hippocampal volume was associated strongly with cell-bound Aß1-40 in blood in patients with AD. The relationships of plasma Aß levels to regional brain atrophy could be attributed to Aß deposition in the brain. A low plasma Aß1-42/Aß1-40 ratio has been linked to increased Aß deposition in the brain parenchyma, which induces brain atrophy, as determined by amyloid positron emission tomography^25^.

### The plasma clusterin level, dementia severity, and the right-side PA score

Several studies have documented increased plasma clusterin levels in patients with AD, although other studies have produced negative findings^22, 31, 32^. Previous studies have also revealed associations between the plasma clusterin level and AD severity and entorhinal cortex atrophy^22, 31, 33^. In our study, the plasma clusterin level was significantly higher in patients with AD than in controls, even after adjustment for multiple confounders. In addition, the association observed between the plasma clusterin level and CDR-SB score was compatible with previous findings^22^. However, we found no association between the MTA rating and the plasma clusterin level. Our statistical power calculation confirmed that this lack of association was not related to the sample size.

We found that the plasma clusterin level was associated significantly with the right-side PA score. In our previous study and in the current sample, right-side PA scores were associated with the NPI agitation/aggression symptoms score^11^.

However, the plasma clusterin level was not correlated with the NPI agitation/aggression or frontal symptoms subscale score. These findings suggest that the plasma clusterin level was correlated with the right-side PA score in patients with AD, but was not associated directly with the NPI agitation/aggression symptoms score. Elizabeta *et al*.^34^ recently found that the plasma/platelet clusterin ratio was associated positively with NPI measures of agitation, apathy, irritability, and motor aberrant behavior in subjects with AD showing BPSD. Further studies are needed to elucidate the potential role of the clusterin level in the development of behavioral symptoms in patients with AD.

### Plasma biomarker and MCI

In patients with MCI, our results showed higher level of plasma Aß1-40 and clusterin than controls, which is in line with previous studies^35, 36^. However, our findings did not show association between plasma biomarkers and clinical symptoms or MR visual rating scores. A recent study showed plasma ratio of Aß1-42/Aß1-40 was stepwise decreased from age-matched controls via MCI to AD and shows a clear correlation with memory scores^37^. Another study showed plasma clusterin level was negatively correlated with cognitive scores of patients with MCI^38^. These discrepancies may relate to the heterogeneous of MCI group, the different diagnostic criteria of MCI, fewer subjects sample size in our group and different measurement tools. From recent review, it showed not all patients with MCI convert to AD reversion of MCI in about 30–50% within 2–5 years follow-up^39^.

### Study limitations

This study has several limitations. First, we did not include all factors potentially confounding plasma Aß data. Previous studies have shown that factors such as age, race, ApoE status, education, and creatinine level may influence plasma Aß levels^40^. In addition, Aß in plasma does not originate exclusively from the brain; it is also the product of amyloid precursor protein metabolism in the skeletal muscle, pancreas, kidney, and liver. Platelets are another important source of Aß1-40 and Aß1-42 in plasma^41^. Thus, the plasma Aß level may only partially reflect Aß in the brain. The methods of plasma Aß and clusterin measurement must also be considered in interpreting our results. We did not measure cell-bound Aß or the platelet clusterin level. Cell-bound Aß may be more closely related than plasma Aß to their physiological counterpart, hippocampal damage^30^. Plasma and platelet clusterin ratios may better correlate with agitation symptoms in patients with AD^34^.

Second, the number of subjects included in the study was not large and age differed significantly between patients with AD and controls. Although the regression analysis was adjusted for age and had sufficient statistical power, we could not completely exclude the confounding effect of age on our results. A study conducted with a large number of age-matched controls might help to build strong evidence for the relationships between plasma biomarkers and clinical symptoms. In addition, as we did not include large number of patients with MCI, our findings are not necessarily applicable to the period before the onset of clinical dementia symptoms. This factor may limit the clinical impact of this study on the early diagnosis of AD.

Third, we did not extensively evaluate whole brain atrophy scores or other regional brain atrophy, such as that occurring in the frontal lobe or subcortical region. This factor may have contributed partially to the lack of a significant association between the plasma clusterin level and MTA score. Furthermore, we could not examine relationships between frontal lobe atrophy and clinical symptoms and plasma biomarkers. Future studies involving whole-brain rating or even voxel-based analysis may improve upon our findings.

## Conclusion

Our results revealed that the plasma Aß1-42/Aß1-40 ratio is correlated with MTA. The mean MTA score was associated significantly with cognitive symptoms. The plasma clusterin level was associated with the severity of AD and right-side PA, which may be related to NPI agitation/aggression symptoms in patients with AD.

## Methods

### Subjects

Subjects with AD were recruited from the outpatient clinic of Taipei Veterans General Hospital between August 2012 and November 2014. The ages between 60 and 90 of both genders with adequate visual and auditory abilities to perform all aspects of the study assessments were included. The diagnosis of probable AD followed the clinical criteria described by the National Institute on Aging–Alzheimer’s Association^42^. Diagnoses were made during a multidisciplinary consensus meeting. All patients underwent a standardized assessment that included the acquisition of data on dementia history, neuropsychological assessment, laboratory tests, and MRI examination. Availability of a reliable caregiver who lives with the subjects or has at least 10 hours/week of contact with the subject was needed. The disease duration was defined as the period between the initial onset of symptoms, reported by caregivers, and the performance of this study. The exclusion criteria was subjects who had significant neurological disease other than AD that may affect cognition, such as Parkinson’s disease, multi-infarct dementia, Huntington’s disease, normal pressure hydrocephalus, brain tumor, progressive supranuclear palsy, seizure disorder, subdural hematoma, multiple sclerosis, or history of significant head trauma followed by persistent neurologic defaults or known structural brain abnormalities were excluded. Controls were recruited among spouses of patients with AD who were included in the study and cognitively healthy patients who presented to the Department of Neurology for the assessment of other disorders. Subjects with histories of major neurological, psychiatric, or severe cardiovascular diseases were excluded from the study. All methods were performed in accordance with the relevant guidelines and regulations. The Institutional Review Board of Taipei Veterans General Hospital approved this study. Written informed consent was obtained from participants and their next of kin or legally authorized representatives.

### Measurement of ApoE gene, plasma Aß and clusterin levels

Genomic DNA was isolated from whole blood using the Gentra Puregene kit (Qiagen, Hilden, Germany), according to the manufacturer’s protocol. The presence of the ε2, ε3, and ε4 alleles of the ApoE gene was determined by assessing the sequences at two single-nucleotide polymorphisms (rs429358 and rs7412)^10, 43^. The ApoE4 gene carrier was defined as one of the alleles containing the ε4 gene.

Blood samples were collected in tubes containing sodium EDTA as an anticoagulant. Following centrifugation, plasma was aliquoted into polypropylene tubes and stored at −80 °C until analysis. Plasma clusterin levels were measured using a Human Clusterin Quantikine ELISA Kit (R&D Systems, Minneapolis, MN, USA) following the manufacturer’s instructions and standard procedures. The plasma Aß peptide assay was performed using the INNO-BIA plasma Aß forms assay (Innogenetics, Ghent, Belgium) based on the multiplex xMAP technique with a LABScan-100 system (Luminex BV, The Netherlands). Each sample was assessed in duplicate. All samples from each participant were measured together on the same plate to avoid interplate variation. The coefficients of variation of the two assays were less than 20%.

### Neuropsychological assessment

Cognitive function was assessed using standardized tests that included several domains. In controls and patients with AD, the MMSE was used to assess global cognition^2^. To evaluate the severity of dementia, the CDR and CDR-SB scores were determined with caregivers’ input^44^. Only patients with AD underwent detailed cognition and NPI studies. To assess memory, we used the delayed recall of 12 items memory test^45^. To examine language function, we used the modified Boston Naming Test^46^. A categorical verbal fluency test was used to evaluate verbal ability and executive control. In this test, the subject named as many fruits as possible within 1 minute^47^. Forward digit span and digit reversal tests were used to assess attention and working memory domain^48^. Finally, the NPI questionnaire was administered to assess the frequency and severity of BPSD^7, 49^. The NPI agitation/aggression, mood and frontal symptoms subscales were used in this study^8^. The NPI agitation/aggression subscale includes agitation/aggression, disinhibition, irritability, and aberrant motor behavior items. The NPI mood subscale includes depression, anxiety, and irritability items. The NPI frontal symptoms subscale includes apathy, disinhibition, irritability, and euphoria items. Each subscale score was calculated by summing item scores (indicating severity) and then multiplying by duration. Trained raters administered all tests in a clinical setting.

### MRI and image analysis

Patients with AD underwent whole-brain MRI (3T DISCOVERY 750; GE) as part of the clinical assessment. First, trans-axial T2-weighted scans (TR/TE = 1130/80 ms, NEX = 2, voxel size = 0.55 × 0.55 × 10 mm^3^), 3D fluid-attenuated inversion recovery (FLAIR) images (TR/TE = 6000/126 ms, inversion time = 1861 ms, NEX = 1, voxel size = 0.56 × 0.56 × 1 mm^3^), and high-resolution sagittal T1-weighted images (TR/TE = 9.1/3.7 ms, NEX = 1, voxel size = 0.5 × 0.5 × 1.0 mm^3^) were acquired. The image analysis and rating procedure has been described in detail previously^11^. In short, image analysis included the visual rating of MTA and PA on T1-weighted images. The MTA in the hippocampus was rated using a 5-point scale^50^. PA in the posterior cingulate, parieto-occipital region, and precuneus was rated using a 4-point scale^51^. Mean MTA and PA scores were calculated for both hemispheres. WMH was evaluated using a scale of age-related white matter change (ARWMC) on axial T2-weighted and 3D-FLAIR images^52^. The 4-point ARWMC scale rates WMH in five brain regions per hemisphere.

### Statistical analysis

All statistical analyses were performed using SPSS (version 21.0; IBM Corporation, Armonk, NY, USA). The skewness and kurtosis of continuous variables, such as age, years of education, BMI, clusterin level, and MMSE score, were within −1 to 1. As skewness values for the plasma Aß1-40 and Aß1-42 levels and the Aß1-42/Aß1-40 ratio exceeded 3, we log transformed these variables for further analysis^53^. Independent two-sample t-tests and X^2^ tests were conducted to compare age, sex, BMI, neuropsychological test scores, plasma Aß and clusterin levels, MTA and PA scores, and ARWMC scores between controls and patients with AD and MCI. Pearson correlation analysis was conducted to study the relationships between plasma biomarkers and age, sex, education, disease duration, BMI, MMSE score, CDR-SB score, and visual rating scores. To explore the associations between plasma biomarkers and MTA, PA, MMSE, cognitive domain, and NPI subscales scores (dependent variables), we used plasma Aß and clusterin levels as independent variables. Age, education, sex, and ApoE4 genetic data were entered as covariates. To assess the relationships between visual rating scores and cognitive functions and neuropsychiatric symptoms, we used regression analysis with MTA and PA scores serving as independent variables, and MMSE, cognitive domain, CDR-SB, and NPI subscale scores serving as dependent variables. Age, education, sex, and ApoE4 genetic data were entered as covariates. Statistical significance was defined as p < 0.05. Post-hoc calculation of the statistical power achieved under the condition α = 0.05 was calculated using the G*Power program (http://gpower.hhu.de/).
